# A taxonomy of childhood pedal cyclist injuries from latent class analysis: associations with factors pertinent to prevention

**DOI:** 10.1186/s40621-021-00366-2

**Published:** 2022-01-24

**Authors:** Joseph Piatt

**Affiliations:** 1 Division of Neurosurgery, Nemours/A I duPont Hospital for Children, 1600 Rockland Road, Wilmington, DE 19803 USA; 2 Departments of Neurological Surgery and Pediatrics, Sidney Kimmel Medical College, Thomas Jefferson University, Philadelphia, PA USA

## Abstract

**Background:**

Studies of pedal cyclist injuries have largely focused on individual injury categories, but every region of the cyclist’s body is exposed to potential trauma. Real-world injury patterns can be complex, and isolated injuries to one body part are uncommon among casualties requiring hospitalization. Latent class analysis (LCA) may identify important patterns in heterogeneous samples of qualitative data.

**Methods:**

Data were taken from the Trauma Quality Improvement Program of the American College of Surgeons for 2017. Inclusion criteria were age 18 years or less and an external cause of injury code for pedal cyclist. Injuries were characterized by Abbreviated Injury Scale codes. Injury categories and the total number of injuries served as covariates for LCA. A model was selected on the basis of the Akaike and Bayesian information criteria and the interpretability of the classes. Associations were analyzed between class membership and demographic factors, circumstantial factors, metrics of injury severity, and helmet wear. Within-class associations of helmet wear with injury severity were analyzed as well.

**Results:**

There were 6151 injured pediatric pedal cyclists in the study sample. The mortality rate was 0.5%. The rate of helmet wear was 18%. LCA yielded a model with 6 classes: ‘polytrauma’ (5.5%), ‘brain’ (9.0%), ‘abdomen’ (11.0%), ‘upper limb’ (20.9%), ‘lower limb’ (12.4%), and ‘head’ (41.2%). Class membership had highly significant univariate associations with all covariates except insurance payer. Helmet wear was most common in the ‘abdomen’ class and least common in the ‘polytrauma’ and ‘brain’ classes. Within classes, there was no association of helmet wear with severity of injury.

**Conclusions:**

LCA identified 6 clear and distinct patterns of injury with varying demographic and circumstantial associations that may be relevant for prevention. The rate of helmet wear was low, but it varied among classes in accordance with mechanistic expectations. LCA may be an underutilized tool in trauma epidemiology.

## Introduction

Because of its morbidity and the potential for prevention by helmet wear, the primary focus of the study of pedal cyclist injuries has been traumatic brain injury (TBI) (Du et al. 2020; Bachynski and Bateman-House 2020 #10560} A secondary focus in the pediatric age group has been abdominal wall and visceral injuries, so-called handlebar injuries (Winston et al. 1998; Nadler et al. 2005). These cardinal injuries do not necessarily occur in isolation. Many children who require hospitalization after a bicycle injury have more than one identified injury. Pedal cyclist injuries are heterogeneous, and exclusive focus on injury to one anatomic region may overlook important associations.

Latent class analysis (LCA) is a method for reducing heterogeneity in qualitative data, like principal component analysis or factor analysis for quantitative data (Agresti 2013). The parameters to be estimated in a latent class model are the probabilities of class memberships and the covariate probabilities conditioned on class memberships. The modeling assumption that permits parameter estimation is that the covariates are independent conditional on class membership. From this assumption and an exercise of Bayes theorem, the expected counts in the covariate matrix can be expressed in terms of model parameters, and estimation can be performed by maximum likelihood methods (Agresti 2013). Because model development requires discretionary decisions about inclusion of covariates and numbers of classes, LCA can seem arbitrary, and interpretation of the classes is not always intuitive. The current exploratory study was carried out to determine whether LCA can bring useful order to a very heterogeneous type of trauma, pedal cyclist injuries among children.

## Methods

The source of the de-identified data for this study was the Trauma Quality Improvement Program (TQIP) of the American College of Surgeons (ACS) for 2017. In that year, 753 trauma centers verified by the ACS participated in TQIP (American College of Surgeons 2017). Centers report data only from hospital admissions. Emergency department discharges and deaths are not included. At the time of hospital discharge, trained and dedicated registrars organize data for submission to a central repository. Risk-stratified outcome reports are returned to the participating institutions for identification of substandard performance. Trauma centers verified by the ACS have met a complex set of requirements for facilities, clinical staffing, performance improvement, and research. Participation in TQIP reflects an additional discretionary institutional commitment above and beyond trauma center verification to collect data about processes of care for quality improvement. In principle, TQIP centers represent the acme of trauma care in the USA, so case mix, injury severities, and lengths of stay may not be representative.

Inclusion criteria were age 18 years or less and primary or secondary International Classification of Diseases, 10th Edition, Clinical Modification (ICD-10-CM) external cause of injury code (Ecode) descriptions that included the text ‘pedal.’ Missing data for age were imputed for infants and toddlers on the basis of weight, as described in a previous study (Piatt 2020). Briefly, TQIP designates age beginning with ‘1.’ There are no observations with age ‘0,’ indicating infancy. Casualties with weight less than 9 kg were assigned age ‘0.’ Casualties with missing age data and weight between 9 and 16 kg were assigned age ‘1.’ Age was then categorized as infant and toddler (less than 3 years), preschool (3 through 5 years), school age (6 through 9 years), teen (10 through 18 years), and missing. There were 7 fields for race, including ‘BLACK,’ ‘WHITE,’ ‘AMERICANINDIAN,’ ‘ASIAN,’ ‘PACIFICISLANDER,’ unknown, and not recorded, and there was a field for Hispanic ethnicity. These fields were not mutually exclusive. They were collapsed into the following categories: ‘Black,’ ‘White,’ ‘Hispanic,’ and ‘other/unknown’ sequentially as follows: All cases coded as ‘BLACK’ were categorized as ‘Black.’ Among the remaining cases, all that were coded for Hispanic ethnicity were categorized as ‘Hispanic.’ Then among the remaining cases, all that were coded ‘WHITE’ were categorized as ‘White.’ The cases that remained were categorized as ‘other/unknown.’ Thus, casualties who were both Black and Hispanic were counted as ‘Black.’ (There were only 19 such cases, 2.1% of the 910 cases originally coded ‘BLACK’ and 1.8% of the 1046 cases originally coded for Hispanic ethnicity.) Payer was categorized as low income (Medicaid and self-pay) or commercial (commercial or other government), and missing (no bill or missing). ICD-10-CM place of injury codes were collapsed into the following categories: road, off-road, domestic or institutional residence, and other/unknown. Injuries occurring in traffic were identified by searching Ecode descriptions for the text ‘nontraffic’ and marking the complement of the set of observations so captured.

Injuries were categorized using the Abbreviated Injury Scale (AIS) (Gennarelli and Wodzin 2006). The AIS system categorizes injuries with a 5- or 6-digit code separated from a single-digit code by a decimal point. The single-digit code designates severity of injury and ranges from 1 for trivial injuries to 6 for nonsurvivable injuries. The 5- or 6-digit code before the decimal point, the ‘PREDOT’ code, indicates a highly specific injury of a particular body region. For example, the PREDOT code ‘858274’ signifies ‘phalange fracture, one of lateral four toes, complete articular, open.’ PREDOT codes were utilized to define binary covariates for the following body regions: major brain (focal or diffuse severity > 2), minor brain (focal or diffuse, severity ≤ 2), skull fracture (severity > 2), facial fracture (any severity), chest visceral (any severity), abdominal visceral (severity > 1), spinal cord (severity > 2), vertebral column (any severity), upper limb (severity > 1), and lower limb (severity > 1). For patients with several injuries coded for the same body category, the most severe injury was definitive. The numbers of injuries ranged from 1 to 38. All cases were coded for at least 1 injury. The 75th percentile was 6. Cases coded for more than 6 injuries were categorized as ‘high injury number.’

Latent class analysis was performed to identify patterns of injury. Entered into the analysis were the body region covariates and the covariate for high injury number. A complete case analysis was performed. The number of latent classes was chosen by the investigator based on the interpretability of the classes supported by the breakpoint in the Akaike and Bayesian information criteria (AIC and BIC). For each observation, the selected model estimated the probabilities of membership in each class, these probabilities summing to unity. Each observation was then assigned a predicted class membership determined by its modal class probability. Univariate associations of class membership with categorical covariates were evaluated by cross-tabulation and Pearson Chi-square tests. Injury Severity Scale (ISS) scores and length of stay (LOS) were both bounded below and highly skewed to the right, so their univariate associates were evaluated with rank-sum tests.

Helmets are designed to protect the head from injury, but helmet wear may be associated with behavior that diminishes or augments the risk of other injuries or their severity. This hypothesis was addressed by analyzing the association between helmet wear and ISS scores and length of stay LOS within latent injury classes. Multivariable associations of helmet wear with metrics of severity of injury were analyzed by nonparametric linear regression within each injury class: Available covariates were entered one by one into a linear regression together with helmet wear and an interaction term. Covariates with significant associations at the *p* < 0.10 level and interaction terms, if significant, were entered into a multivariable regression and then eliminated stepwise. The final model consisted of helmet wear plus covariates and interactions retaining significance after a Bonferroni correction for the 12 final models. Parameter estimates for helmet wear are reported. These estimates reflect the numerical effects on ISS and LOS associated with helmet wear. Because ISS and LOS are both greatly skewed to the right, confidence intervals were calculated by Poisson bootstrap with 10,000 repetitions.

A risk of type 1 error less than 0.05 was considered significant, and all hypothesis tests were two-tailed. Data were organized and analyzed in R (R Foundation, Vienna, Austria) using RStudio with the poLCA package (Linzer et al. 2011; RStudio T. Rstudio 2020). This project was judged not to be human subjects research by the Nemours Delaware Valley Institutional Review Board.

## Results

There were 6151 cases of injured cyclists. Characteristics of this sample are presented in Table 1. The overall rate of helmet wear was 18%. The mortality rate was 0.5%.

**Table 1:** Characteristics of the sample (*N* = 6151)

*Sex*	
Male	4874 (79%)
Female	1276 (21%)
Data missing	1
*Age group*	
Infants and toddlers	84 (1.4%)
Preschoolers	534 (8.7%)
School age children	1681 (27%)
Teens	3851 (63%)
Data missing	1
*Race and ethnicity*	
Black	910 (15%)
Hispanic	1027 (17%)
other	507 (8%)
White	3584 (59%)
data missing	123 (2%)
*Insurance*	
Commercial	3006 (50%)
Low income	2960 (49%)
Medicare	39 (1%)^a^
Data missing	146 (2%)
*Place of injury*	
Road	3620 (59%)
Off-road	743 (12%)
Residential	595 (10%)
Unknown	1193 (19%)
*Circumstances*	
In traffic	2488 (40%)
Elsewhere	3663 (60%)

^a^Not analyzed further

**Fig. 1:**
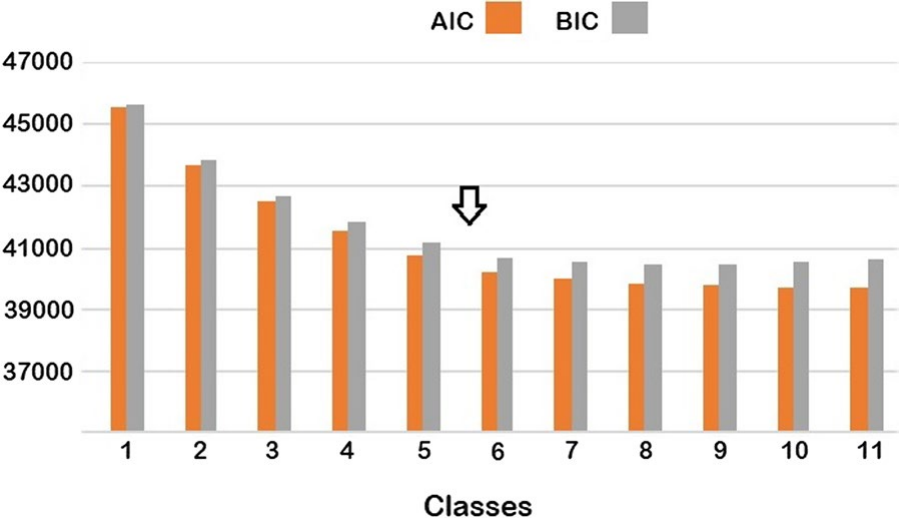
Akaike and Bayesian information criteria for latent class models. These metrics both fell in a monotonic fashion with increasing class numbers. Note the breakpoint in the fall between 5 and 6 classes (arrow). Beyond this point increasing numbers of classes yielded no improvements in the information criteria

A model with 6 latent classes was selected on the basis of the AIC and BIC for models with 1–11 classes. AIC and BIC fell steadily—reflecting better model fit—as the number of classes increased to 6, but there was very little decline beyond that number. Figure 1 illustrates this breakpoint in the fall of AIC and BIC with class number. The 6 classes were labeled ‘polytrauma,’ ‘brain,’ ‘abdomen,’ ‘upper limb,’ ‘lower limb,’ and ‘head.’ Class membership and probabilities of injury conditional on class membership are presented in Table 2. The model had good fit with a deviance of 1018.783 on 1976 degrees of freedom. The designations of the classes are self-explanatory with the exception of the ‘head’ class. This class was so designated because of the relatively high probability of minor brain injury and facial injury, but it is notable as well for high injury number. Membership in the ‘head’ class was the most prevalent by far, and injury severity in this class was the lowest.

**Table 2:** Six-class LCA model of patterns of pedal cyclist injury

	Polytrauma	Brain	Abdomen	Upper limb	Lower limb	Head
Estimated class probabilities	0.06	0.09	0.17	0.20	0.12	0.37
Predicted class membership (%)	5.5	9.0	11.0	20.9	12.4	41.2
Major brain	0.32	1.00	0.01	0.00	0.00	0.00
Minor brain	0.26	0.00	0.02	0.00	0.02	0.53
Skull fracture	0.26	0.40	0.00	0.00	0.00	0.07
Face fracture	0.39	0.16	0.02	0.01	0.00	0.23
Chest visceral	0.57	0.02	0.06	0.01	0.01	0.01
Abdominal visceral	0.31	0.00	0.55	0.00	0.02	0.01
Spinal cord	0.03	0.00	0.00	0.00	0.00	0.00
Vertebral	0.24	0.01	0.03	0.00	0.01	0.02
Upper limb	0.37	0.06	0.04	1.00	0.06	0.09
Lower limb	0.47	0.03	0.00	0.00	1.00	0.04
High injury number	0.99	0.28	0.01	0.01	0.05	0.15

‘Estimated class probabilities’ refers to the model-estimated probability that a randomly selected casualty belongs in one or another latent class. The model also calculates for each individual casualty a probability of membership in each class. The modal probability of class membership determines class membership for further analysis. ‘Predicted class membership’ indicates the fraction of casualties assigned to each latent class. Finally, the model estimates probabilities of the various injures conditional on class membership

Class membership had highly significant associations with age group, sex, race and ethnicity, helmet wear, place of injury, injury in traffic, injury number, ISS, LOS, and mortality (all *p* values < 0.00001). Univariate associations with selected covariates are presented in Table 3. The only covariate in the data set not associated with class membership was payer (*p* = 0.1587). Injuries of all types were most numerous among teens, but the polytrauma and brain classes accounted for larger fractions of teen injuries than in other age groups. Preschool children were overrepresented in the upper limb class, and although there were few injured infants and toddlers in the sample, they were overrepresented in the lower limb class. There were disproportionately few females in the polytrauma class and disproportionately more in the upper limb class. White children accounted for more than their share of abdominal injuries. Black children were underrepresented in the upper limb class and overrepresented in the lower limb class. Children wearing helmets were relatively less common in the polytrauma and head classes and relatively more common in the abdomen class. The polytrauma and head classes accounted for larger fractions of road injuries than of injuries elsewhere, and the abdomen and upper limb classes accounted for smaller fractions. Residential injuries were markedly underrepresented in the polytrauma and brain classes. Injuries in traffic were numerically less common than injuries not in traffic, but they accounted for 4/5ths of the polytrauma class. They were notably underrepresented in the abdomen and upper limb classes. Injury number, ISS scores, LOS, and mortality were far greater in the polytrauma class than in other classes, as expected.

**Table 3:** Univariate associations of class membership with covariates pertinent to prevention efforts

	Polytrauma	Brain	Abdomen	Upper limb	Lower limb	Head
*Age group*						
Infant and toddler	5 (6%/1%)	5 (6%/1%)	1 (1%/0%)	18 (21%/1%)	27 (32%/4%)	29 (34%/1%)
Preschool	13 (2%/4%)	36 (7%/6%)	22 (4%/3%)	198 (37%/15%)	63 (12%/8%)	202 (37%/8%)
School age	46 (3%14%)	116 (7%/21%)	216 (12%/32%)	429 (26%/33%)	176 (10%/23%)	698 (42%/28%)
Teen	274 (7%/81%)	398 (10%/72%)	439 (11%/65%)	640 (17%/50%)	495 (13%/65%)	1605 (42%/63%)
*Sex*						
Female	52 (4%/15%)	105 (8%/19%)	123 (10%/18%)	337 (26%/26%)	143 (11%/19%)	516 (40%/20%)
Male	286 (6%/85%)	449 (9%/81%)	555 (11%/82%)	948 (19%/74%)	618 (13%/81%)	2018 (41%/80%)
*Race/ethnicity*						
Black	56 (6%/17%)	62 (7%/11%)	84 (9%/13%)	142 (16%/11%)	143 (16%/19)	423 (46%/17%)
Hispanic	64 (6%/20%)	100 (10%/18%)	96 (9%/14%)	242 (24%/19%)	126 (12%/17%)	399 (39%/16%)
Other	24 (5%/7%)	52 (10%/10%)	42 (8%/6%)	116 (23%/10%)	54 (11%/8%)	219 (43%/9%)
White	182 (5%/56%)	333 (9%/61%)	442 (12%/67%)	767 (21%/61%)	424 (12%/57%)	1436 (40%/58%)
*Payer*						
Commercial	168 (6%/52%)	273 (9%/51%)	366 (12%/55%)	621 (21%/49%)	364 (12%/49%)	1214 (40%/50%)
Low income	156 (5%/48%)	266 (9%/49%)	297 (10%/45%)	635 (21%/51%)	381 (13%/51%)	1225 (41%/50%)
*Helmet wear*						
Yes	48 (4%/14%)	51 (5%/9%)	187 (17%/28%)	263 (24%/20%)	137 (12%/18%)	426 (38%/17%)
No	290 (6%/86%)	504 (10%/91%)	491 (10%/72%)	1022 (20%/80%)	624 (12%/82%)	2108 42%/(83%)
*Place of injury*						
Road	295 (8%/87%)	377 (10%/68%)	318 (9%/47%)	533 (15%/41%)	494 (14%/65%)	1603 (44%/63%)
Off-road	20 (3%/6%)	55 (7%/10%)	114 (15%/17%)	153 20%/12%)	86 (12%/11%)	316 (43%/12%)
Residential	4 (1%/1%)	35 (6%/6%)	65 (11%/10%)	208 (35%/16%)	75 (13%/10%)	208 (35%/8%)
Unknown	19 (2%/6%)	88 (7%/16%)	181 (15%/27%)	392 (33%/31%)	106 (9%/14%)	407 (34%/16%)
*Injury in traffic*						
Yes	262 (11%/78%)	240 (10%/43%)	194 (8%/29%)	332 (13%/26%)	361 (15%/47%)	1099 (44%/43%)
No	76 (2%/22%)	315 (9%/57%)	484 (13%/71%)	953 (26%/74%)	400 (11%/53%)	1435 (39%/57%)
Number of injuries	10 [8–13]	5 [3–7]	2 [1–3]	2 [1–2]	2 [1–4]	3 [2–5]
Length of stay, days (median, IQR)	5 [3–9]	3 [2–4]	3 [2–4]	1 [1–2]	3 [2–4]	1 [1–2]
ISS (median, IQR)	17 [14–29]	14 [10–17]	9 [5–10]	4 [4–5]	9 [5–9]	3 [1–5]
Mortality	5.9%	0.9%	0.1%	0.0%	0.3%	0.0%

All associations were highly significant with *p* < 0.00001, except for payer (*p* = 0.1587). Raw counts and row/column percentages

IQR, interquartile range

The only preventative measure recorded in this data set was helmet wear. As has been seen, helmet wear was strongly associated with class membership on a univariate level. The associations of helmet wear with ISS score and LOS within classes are presented in Table 4. The effects were very small. No interaction of other covariates with helmet wear retained significance after Bonferroni correction. The effect of helmet wear was not significant for any measure of injury severity in any injury class.

**Table 4:** Estimation of the effect of helmet wear on Injury Severity Scale (ISS) score and length of stay (LOS) for each latent class with 95% confidence intervals

Latent class	ISS effect (points)	LOS effect (days)
Polytrauma	− 1.25 (− 4.87 to 2.69)	− 0.52 (− 3.20 to 2.91)
Brain	0.36 (− 1.70 to 2.64)	0.23 (− 0.84 to 1.35)
Abdomen	0.64 (− 0.39 to 1.75)	− 0.03 (− 1.16 to 1.32)
Upper limb	0.14 (− 0.02 to 0.30)	0.02 (− 0.11 to 0.15)
Lower limb	0.40 (− 0.42 to 1.28)	− 0.34 (− 0.71 to 0.04)
Head	− 0.05 (− 0.42 to 0.33)	− 0.03 (− 0.16 to 0.11)

For example, helmet wear was associated with a diminution of ISS by 1.25 points

None of these effects was significant

## Discussion

Pedal cyclist injuries are heterogeneous with respect both to their anatomic distributions and to their circumstantial mechanisms. LCA has been applied to police reports of bicycle crashes. Yasmin and Eluru used a latent class method to segment traffic analysis zones in Montreal and Toronto on the basis of cyclist injury counts and a variety of local demographic and geographic characteristics (Yasmin and Eluru 2016). Based on 2 years of Italian data, Prati et al. identified 19 latent classes of bicycle crashes from a host of crash characteristics such as weather conditions, road topography, and cyclist and motorist actions (2017). Factors contributing to the severity of the injuries were then analyzed within classes. Sivasankaran and Balasubramanian conducted a similar study of the circumstances of bicycle injuries in the Indian state of Tamil Nadu over a 9-year period (2020). They identified factors associated with injury severity in certain latent classes that were of no significance in the larger unsegmented sample. Likewise, Lin and Fan studied crashes between cyclists and motor vehicles in North Carolina over a 7-year period (Lin and Fan 2021). Within each of the 7 homogeneous latent classes, the effects of selected factors on severity of injury were estimated with a partial proportional odds model.

As far as the author is aware, the current investigation is the first attempt to apply LCA to clinical data from pedal cyclist injuries. From the TQIP sample, 6 conceptually clear and distinct classes emerged. The potential value of injury pattern recognition is apparent: Prevention of cyclist head injury is a pressing concern, but prevention of isolated head injuries may require different measures than prevention of head injuries associated with pulmonary contusions, ruptured viscera, and long bone fractures. The observations of the current study do not lead directly to prevention initiatives, but they might inform and direct such initiatives. For example, classes representing musculoskeletal injuries accounted for more than a third of the cases in the TQIP sample. Musculoskeletal injuries might thus be considered a worthy target for prevention, but any intervention must take into account the differences between upper limbs and lower limbs. Upper limb injuries were relatively common among preschool children and among females, and relatively less common among Black children and among injuries sustained in traffic and on the road. Lower limb injuries were relatively common among infants and toddlers and among Black children. Ten percent of the TQIP sample fell into the abdomen class. Planning for prevention of abdominal injuries might consider their prevalence among school age and teen White children cycling off-road and out of traffic.

The only preventative measure recorded in this data set was helmet wear. There is a large body of evidence, reviewed and summarized in numerous publications, to the effect that helmets prevent head injury (Du et al. 2020; Bachynski and Bateman-House 2020; American College Safety 2014; Dagher et al. 2016; Persaud et al. 2012; Strotmeyer et al. 2020; Meehan et al. 2013). Consistent with these data, the current study found helmet wear lowest in the ‘polytrauma’ and ‘brain’ classes and highest in the ‘abdomen’ class. An association of helmet wear with cyclist behavior has been postulated as well—for both better and worse (Michael et al. 2017; Hoye et al. 2020; Schleinitz et al. 2018). This hypothesis was explored in the current study by analyzing the association of helmet wear with ISS scores and LOS within classes. There were no supportive findings. Beyond mechanical protection of the head, no behavioral effect of helmet wear was detected in these data.

The most salient limitation of the current study is the impossibility of a control sample of uninjured cyclists. An uninjured control group would have permitted more direct analysis of behavioral questions, for instance, whether the prevalence of helmet wear is different between uninjured cyclists and cyclists in the lower limb class. An alternate approach was taken in analysis of the effect of helmet wear on injury severity within classes. This analysis was negative. It may have been underpowered particularly to detect confounding interactions, but all effect sizes were small. Another limitation was the absence from the sample of casualties who did not require hospitalization. Excluded, for example, were dental injuries. The construction of this latent class model involved numerous discretionary decisions that might be questioned or criticized. The model examined here is not the only possible model, nor is it objectively the best model that might have been developed from the data sample. It was adopted because of its satisfactory fit to the data and its ready interpretability. Whether LCA developed from a different sample of injured pediatric cyclists would converge on the same classes is unknown. An LCA of injured adult cyclists would not be expected to identify the same classes.

## Conclusions

In this sample taken from a year of TQIP data, LCA identified 6 clear and distinct patterns of injury with varying demographic and circumstantial associations that may be relevant for prevention. The rate of helmet wear was low, but it varied among classes in accordance with mechanistic expectations. Helmet wear had no detectable effect on injury severity within classes. LCA may be an underutilized tool in trauma epidemiology.

## Data Availability

The data utilized in this project are available from the author upon reasonable request, contingent on explicit permission from the American College of Surgeons.

## Abbreviations

ACS: American College of Surgeons
AIC: Akaike information criterion
AIS: Abbreviated Injury Scale
BIC: Bayesian information criterion
ISS: Injury Severity Scale
LCA: Latent class analysis
LOS: Length of stay
TQIP: Trauma Quality Improvement Program
